# Spatial transcriptome analysis provides insights of key gene(s) involved in steroidal saponin biosynthesis in medicinally important herb *Trillium govanianum*

**DOI:** 10.1038/srep45295

**Published:** 2017-03-28

**Authors:** Pradeep Singh, Gagandeep Singh, Abhishek Bhandawat, Gopal Singh, Rajni Parmar, Romit Seth, Ram Kumar Sharma

**Affiliations:** 1 Biotechnology Department, CSIR-Institute of Himalayan Bioresource Technology, Palampur, Himachal Pradesh, 176061, India

## Abstract

*Trillium govanianum*, an endangered medicinal herb native to the Himalaya, is less studied at the molecular level due to the non-availability of genomic resources. To facilitate the basic understanding of the key genes and regulatory mechanism of pharmaceutically important biosynthesis pathways, first spatial transcriptome sequencing of *T. govanianum* was performed. 151,622,376 (~11.5 Gb) high quality reads obtained using paired-end Illumina sequencing were *de novo* assembled into 69,174 transcripts. Functional annotation with multiple public databases identified array of genes involved in steroidal saponin biosynthesis and other secondary metabolite pathways including brassinosteroid, carotenoid, diterpenoid, flavonoid, phenylpropanoid, steroid and terpenoid backbone biosynthesis, and important TF families (bHLH, MYB related, NAC, FAR1, bZIP, B3 and WRKY). Differentially expressed large number of transcripts, together with CYPs and UGTs suggests involvement of these candidates in tissue specific expression. Combined transcriptome and expression analysis revealed that leaf and fruit tissues are the main site of steroidal saponin biosynthesis. In conclusion, comprehensive genomic dataset created in the current study will serve as a resource for identification of potential candidates for genetic manipulation of targeted bioactive metabolites and also contribute for development of functionally relevant molecular marker resource to expedite molecular breeding and conservation efforts in *T. govanianum*.

*Trillium govanianum* Wall. ex D. Don (Family: Melanthiaceae), prevalently known as “Nag chhatri” or “Satva”, is an endangered perennial herb with multiple therapeutic properties. It is the only species of the genus *Trillium*, native to the Himalaya representing scattered distribution from Nanga Parbat to Namcha Barwa at an altitude range of 2700 m to 4000 m^1^,^2^. It is an allotetraploid (2n = 4× = 20) between the genus *Trillium* and *Daiswa*, having a very large genome size of 67.125 Gb (1C = 68.64 pg)^3^,^4^. The species is characterized by the presence of three leaves in a whorl just beneath a trimerous flower emerging from a 15–30 cm long solitary stem and bright red berry like globose fruit at the top, containing numerous seeds. Flowering has been reported to occur during the months of May to July followed by seed set in October^3^. The short and stout rhizome along with adventitious roots has been used in the Indian system of medicine as “folk medicine” (http://www.medicinalplants.in/folksearchpage) to cure menstrual and other reproductive disorders^5^, general body weakness^6^, dysentery^2^, headache and fever^7^, besides its use as an anthelmintic for livestock^8^. Great medicinal importance coupled with high commercial value (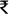
15,000 to 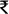
20,000 per kg raw botanical drug) in the international markets^9^ has led to quantum extraction of *T. govanianum* before the maturation of seeds. This has resulted in rapid depletion of its natural populations and makes this species endangered in the Himalaya^10^,^11^.

Medicinal and therapeutic importance of the species is due to the occurrence of steroidal saponins, one of the most structurally diverse and extensively distributed secondary metabolites in plants^12^. The diverse nature, number and linkage pattern of sugar moieties in aglycone skeleton contributes to the broad range of biological and pharmacological functions of steroidal saponins. Although, a large number of steroidal saponins have been identified in the genus *Trillium*^13^,^14^,^15^, only four spirostanol saponin (govanoside A, borassoside E, pennogenin, diosgenin) have been isolated from the *T. govanianum,* so far. Among these, diosgenin that accumulates in rhizome as “Trillarin” is considered as the main bioactive constituent of *T. govanianum*^16^,^17^. Globally, diosgenin is used as anti-cancerous and anti-aging agent, besides its use as precursor for the preparation of many steroidal drugs^18^. Interestingly, *T. govanianum* accumulates almost triple diosgenin content (~6.0%) compared to other explored medicinal plant species, namely *Asparagus* spp., *Chlorophytum* spp., *Dioscorea* spp. and *Trigonella* spp^19^.

Steroidal saponins are mainly synthesized from squalene through isopentenyl diphosphate (IPP) and dimethylallyl diphosphate (DMAPP) via cytosolic mevalonate (MVA) and plastidial 2-C-methyl-D-erythritol 4-phosphate (MEP) pathways, respectively. The cyclization of 2,3-oxidosqualene synthesized from squalene is the first diversifying step for the biosynthesis of steroidal and triterpenoid saponins catalyzed by oxidosqualene cyclases (OSCs). In plants, cycloartenol synthase and lanosterol synthase mediates the cyclization of 2,3-oxidosqualene to synthesize steroidal saponins^20^,^21^,^22^. Further, several hydroxylation and glycosylation steps catalyzed by CYP450s and UGTs respectively, contributes to the structural and functional diversification of various steroidal saponins^23^.

Despite its vast commercial and medicinal importance, merely 12 nucleotides and 10 protein sequences (http://www.ncbi.nlm.nih.gov) have been reported in *T. govanianum*. Considering limited genomic information coupled with genome complexity (polyploidy and large genome size), elucidation of complex steroidal saponin biosynthetic pathway^24^ would be very challenging at the genome level. RNA sequencing (RNA-Seq) with the availability of various cost effective NGS platforms has been proved to be an efficient tool for genome-wide transcriptome profiling and elucidation of important candidates involved in complex biosynthetic pathways, irrespective of genome complexity even in case of non-model plant species^25^.

In the current study, for identification of key genes involved in complex steroidal saponin pathway, a comprehensive spatial transcriptome of endangered *T. govanianum* has been sequenced using Illumina GAIIx platform. Efforts were also made to identify potential transcription factors, CYPs and UGTs that play key role in regulation and diversification of secondary metabolites. Current findings provide first genome-wide transcriptional insights of steroidal saponin gene functions and their spatial differential expression in rhizome, stem, leaf and fruit tissues of *T. govanianum.* Futuristically, outcome of current findings will serve as a resource to expedite cutting edge research for up-scaling of targeted secondary metabolite production through genetic engineering of *T. govanianum*, besides its use for creation of functionally relevant molecular marker resource to assist population genetics and conservation studies.

## Results

### RNA sequencing and *de novo* assembly

**Figure 1:**
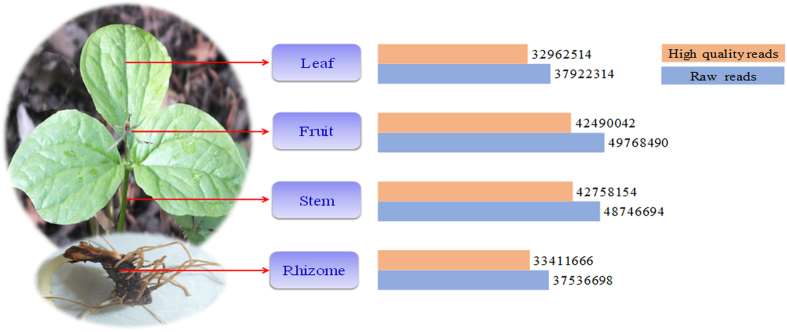
Details of tissue used for spatial transcriptome analysis vis-à-vis raw and high quality reads obtained in *T. govanianum*.

Wider applicability of NGS technologies, including discovery of novel genes, tissue specific expression analysis and sequence based molecular marker resource creation provides excellent opportunity to dissect complex biosynthetic pathways and enables understanding genomics of various non-model plants^25^. We used Illumina GAIIx to sequence the cDNA libraries of rhizome, stem, leaf, and fruit tissues for elucidating secondary metabolites biosynthesis and understanding their spatial expression pattern in *T. govanianum.* The paired-end (PE) sequencing of four different libraries resulted into 173,974,146 raw reads, ranging from 37 to 49 million for each library (Fig. 1). Quality filtering after removal of adaptor sequences, ambiguous and low quality reads, 151,622,376 (~11.5 Gb) high quality reads were obtained. *De novo* assembly of clean reads using CLC Genomics Workbench, resulted into 69,174 non-redundant (NR) assembled transcripts (~27.74 Mb). The length of the transcripts varied from 174 to 35,947 bp with an average of 401 bp, N50 (412 bp) and GC content of 46.6% (Supplementary Table S1). The raw reads generated from Illumina GAIIx sequencing of all the four tissues were deposited at National Centre for Biotechnology Information (NCBI) Sequence Read Archive (SRA) database with accession number: SRP090722 under the Bioproject- PRJNA345073.

### Functional annotation and classification

**Figure 2:**
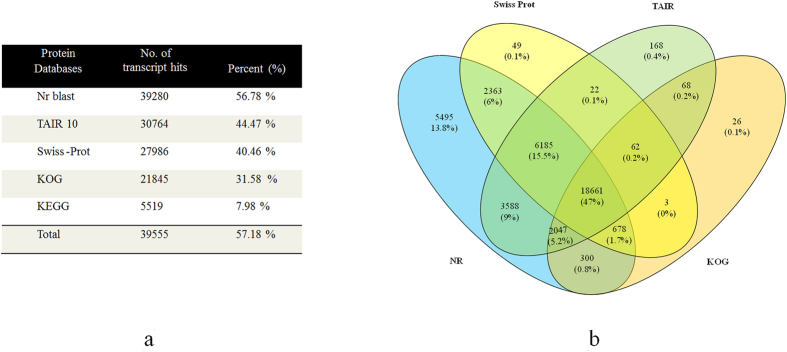
Statistics overview of functional annotations. (**a**) Functional annotations of transcripts with various public protein databases, (**b**) Venn diagram showing functional annotation details of transcripts with NR, TAIR10, Swiss-Prot and KOG databases.

Functional characterization of transcriptomic data provides a global overview of biological processes, molecular functions and abundance of biosynthesis pathways. Being a non-model plant, to get the optimum annotations, assembled transcripts of *T. govanianum* were aligned with five public protein databases. Annotations of 69,174 transcripts identified 39,280 (56.78%; NCBI’s nr), 30,329 (43.84%; TAIR10), 27,986 (40.46%; Swiss-Prot) and 21,845 (31.58%; KOG) transcripts showing putative functions (Fig. 2a; Supplementary Table S2). Interestingly, 5,495, 168, 49 and 26 transcripts were uniquely annotated to nr, TAIR10, Swiss-Prot and KOG, respectively (Fig. 2b). Due to the non-availability of genomic and transcriptomic resources in targeted species, 29,169 (42.82%) transcripts could not be annotated to any of the searched databases.

**Figure 3:**
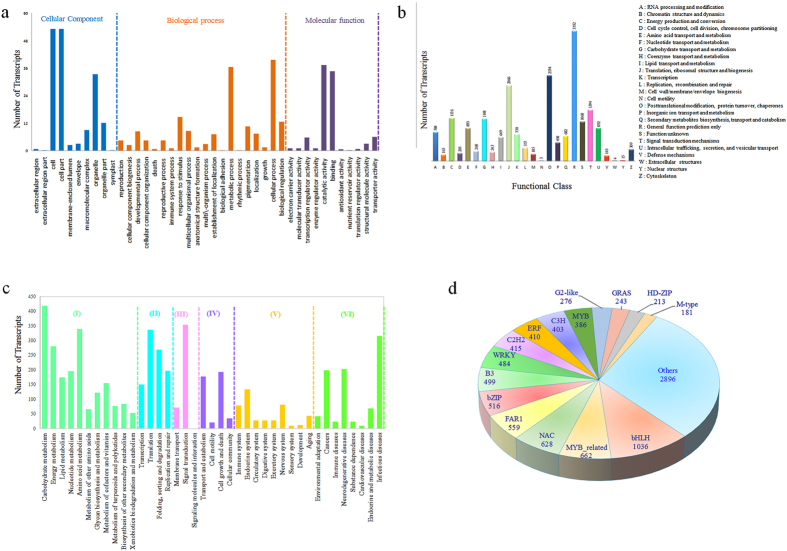
Function annotation classifications. (**a**) Gene Ontology (GO) classification summarized into, cellular component, biological process and molecular function categories; (**b**) EuKaryotic Orthologous Groups (KOG) classification of transcripts into 25 categories; (**c**) Kyoto Encyclopedia of Genes and Genomes (KEGG) classification details of six main categories, I: Metabolism, II: Genetic information processing, III: Environmental information processing, IV: Cellular processes, V: Organismal systems, (VI) Human diseases, (**d**) Classification of transcripts into major transcription factor families.

Gene ontology (GO) has been widely used for functional analysis and inferring biological significance of genomic and transcriptomic datasets^26^. A total of 28,838 transcripts having 12,938 unique TAIR IDs were assigned 44,043 GO terms, wherein 15,679 terms were categorized into molecular function, 16,209 into biological process and 12,155 into cellular component. Among the molecular function, GO terms related to catalytic activity (37.4%) and binding (34.7%) were most abundant, followed by transporter activity (6.0%) and transcription regulator activity (5.7%). Among the biological process, cellular process (39.7%) and metabolic process (36.5%) were the most represented followed by response to stimulus (14.7%), biological regulation (12.5%) and pigmentation (10.6%). While in cellular component, cell (53.2%) and cell part (53.2%) recorded ample representation followed by organelle (33.3%) and organelle part (12.1%) (Fig. 3a).

Further, to assess the competence of *de novo* assembly and effectiveness of the annotation process, alignment of transcripts with KOG database annotated 21,845 transcripts. Of these, 19,763 transcripts were uniquely classified into 25 KOG categories, while remaining 2,082 were annotated with multiple KOG functions, hence cannot be classified to any category. The general function prediction with 3,552 (17.9%) transcripts was evident as the major KOG category, followed by post-translational modification, protein turnover, chaperones (2,334 transcripts, 11.8%) and translation, ribosomal structure and biogenesis (2,066 transcripts, 10.45%). Interestingly, a total of 682 (3.48%) transcripts were assigned to secondary metabolites biosynthesis, transport and catabolism category (Fig. 3b).

KEGG annotations provide background of active metabolic processes within an organism, hence, enables further understanding of the biological function of the transcripts^27^. To elucidate active biosynthesis pathways in *T. govanianum,* annotation of NR data with KEGG database discovered 5,519 transcripts comprising of 3,553 unique KO identifiers. Of these, 3,752 transcripts with 2,338 unique KO identifiers were assigned to six main categories representing 332 biological pathways. The highest number of KO identifiers were involved in metabolism (1,962) followed by genetic information processing (952), human diseases (841), organismal systems (482), environmental information processing (425) and cellular processes (425). Pathways with largest number of KO identifiers were carbohydrate metabolism (418), signal transduction (353), amino acid metabolism (340), translation (336) and infectious diseases (315). Interestingly, significant number of genes involved in the biosynthesis of other secondary metabolites (84), metabolism of terpenoids and polyketides (77) were also identified (Fig. 3c).

Transcription factors (TFs) are major regulatory elements, playing significant role in gene expression, plant secondary metabolism and response to environmental stress by binding to specific cis-regulatory elements of the promoter regions^28^. TF families, including ARF, bHLH, bZIP, MYB, NAC, and WRKY were reported to be involved in regulation of secondary metabolites, abiotic and biotic stress responses in many plant species^29^,^30^. In our study, a total of 9,807 (14.17%) transcripts were assigned to 58 TF families. Among these, bHLH (1,036), MYB related (662), NAC (628), FAR1 (559), bZIP (516), B3 (499) and WRKY (484) were found to be most abundant, possibly involved in the regulation of various physiological processes and biochemical pathways in *T. govanianum* (Fig. 3d; Supplementary Table S3).

### Tissue specific differential gene expression

**Figure 4:**
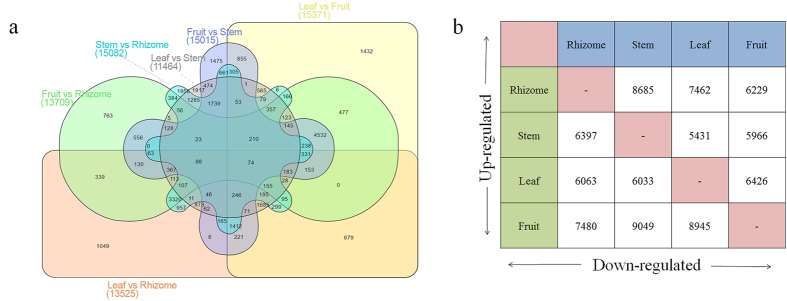
Differential expressed genes (DEG) in *T. govanianum*. (**a**) Venn diagram showing common and unique genes (**b**) Pair wise comparison of up and down-regulated genes in different tissues.

To understand the key putative regulators involved in steroidal saponin biosynthesis, tissue specific gene expression was measured using edgeR program. The transcripts with log_2_ fold change (FC) >2 and *p*-value < 0.05 were considered as differentially expressed genes (DEGs). Pair-wise comparison of transcripts in different tissues resulted into13,525 DEGs in leaf vs rhizome (7,462 up-regulated and 6,063 down-regulated), 15,371 in leaf vs fruit (8,945 up-regulated and 6,426 down-regulated), 11,464 in leaf vs stem (5,431 up-regulated and 6,033 down-regulated), 13,709 in fruit vs rhizome (6,229 up-regulated and 7,480 down-regulated), 15,015 in fruit vs stem (5,966 up-regulated and 9,049 down-regulated) and 15,082 in stem vs rhizome (8,685 up-regulated and 6,397 down-regulated). Further, a total of 1,049, 1,432, 1,917, 763, 1,475 and 1,956 unique DEGs were obtained in leaf vs rhizome, leaf vs fruit, leaf vs stem, fruit vs rhizome, fruit vs stem and stem vs rhizome, respectively (Fig. 4a,b).

#### Cytochrome P450s

Cytochrome P450s (CYPs) are the members of monooxygenases superfamily and known to be involved in the diversification of a wide range of plant secondary metabolites, including lignin, terpenoids, sterols, fatty acids and saponins^31^. A total of 108 CYP genes corresponding to 275 transcripts classified under 34 families were identified in *T. govanianum* (Supplementary Table S4). Among the various CYPs, CYP71 family (predominantly CYP71A5) was the most abundant. Interestingly, CYP51G1, a type of multifunctional oxidases known to be involved in sterol and steroid biosynthesis was also identified^32^. In total, 87 CYP genes (202 transcripts) related to 30 families were found to be differentially expressed at least in one pair-wise comparison. The tissue specific expression of these CYP genes revealed that 36, 21, 17 and 13 genes were highly expressed in leaf, stem, fruit and rhizome, respectively (Supplementary Figure S1a; Supplementary Table S5).

#### UDP-glycosyltransferases

Uridine diphosphate-glycosyltransferases (UGTs) belong to glycosyltransferase (GT) family1, which catalyzes transfer of glycosyl group from UTP-sugar to various metabolites, including steroidal saponins. Usually, UGTs are involved during the last stages of secondary metabolite biosynthesis, thus having a significant role in diversification, stability and modification of biologically active end products^33^. A total of 58 UGTs (173 transcripts), classified under 20 families were identified in *T. govanianum* NR data (Supplementary Table S4). UGT73 and UGT85 were the most predominant families, represented by 62 and 17 transcripts, respectively. A total of 125 transcripts representing 49 UGTs (18 families) were differentially expressed at least in one pair-wise comparison. The tissue specific expression of 49 UGT genes revealed that 15, 14, 10 and 10 genes were highly expressed in stem, fruit, leaf and rhizome, respectively (Supplementary Figure S1b; Supplementary Table S5). Sterol 3-beta-glucosyltransferases (UGT80B1), an important enzyme of sterol glycoside and steroidal saponins biosynthesis had shown higher expression in fruit^34^,^35^.

#### Secondary metabolic pathway analysis

**Figure 5:**
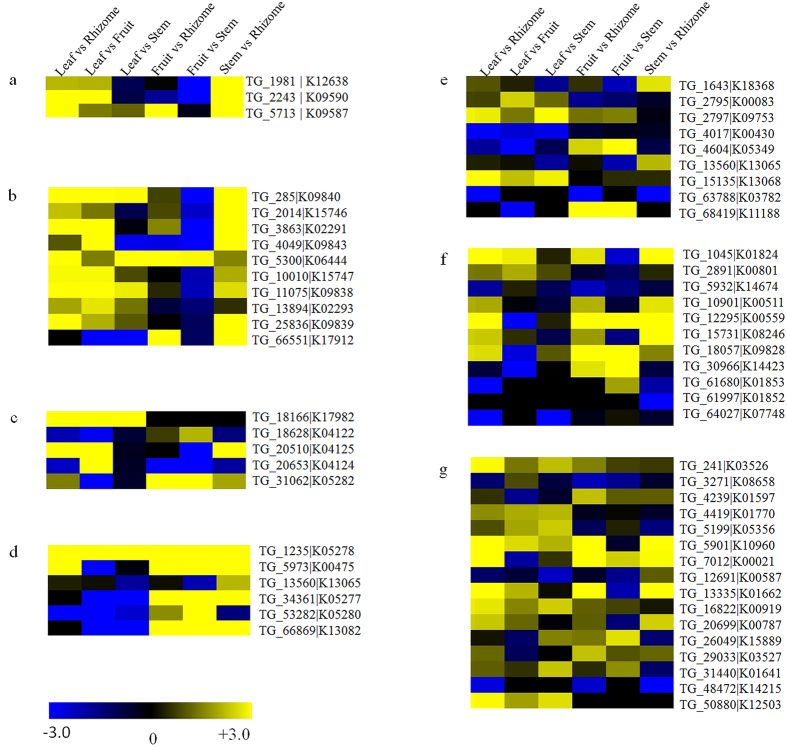
Heat map representing expression dynamics of genes involved in seven secondary metabolite biosynthesis pathways. (**a**) Brassinosteroid biosynthesis (**b**) Carotenoid biosynthesis (**c**) Diterpenoid biosynthesis (**d**) Flavonoid biosynthesis (**e**) Phenylpropanoid biosynthesis (**f**) Steroid biosynthesis (**g**) Terpenoid backbone biosynthesis.

Metabolic pathway analysis enables us to understand the interactions of genes in particular pathway and their related biological functions^36^. A total of 27 pathways (206 transcripts) related to secondary metabolite biosynthesis were identified from the KEGG database (Supplementary Table S6). The identification of these pathways helped us in analyzing secondary metabolite biosynthesis in *T. govanianum*. Out of these, seven major pathways, namely brassinosteroid, carotenoid, diterpenoid, flavonoid, phenylpropanoid, steroid and terpenoid backbone biosynthesis were well represented in our data. Out of 141 transcripts involved in these pathways, 78 recorded tissue specific differential expression (Fig. 5; Supplementary Table S5). The genes involved in brassinosteroid and carotenoid pathways were highly expressed in leaf and stem tissues (Fig. 5a,b), while key genes involved in flavonoid pathway were found to be highly expressed in fruit, followed by stem (Fig. 5d). Interestingly, most of the genes involved in terpenoid backbone biosynthesis recorded higher expression in leaf (Fig. 5g), while, genes involved in steroid pathway found to be highly expressed in rhizome and fruit (Fig. 5f). Nonetheless, genes related to diterpenoid and phenylpropanoid pathways showed variable expression in rhizome, stem, leaf and fruit (Fig. 5c & e).

### Steroidal saponin pathway genes

**Figure 6:**
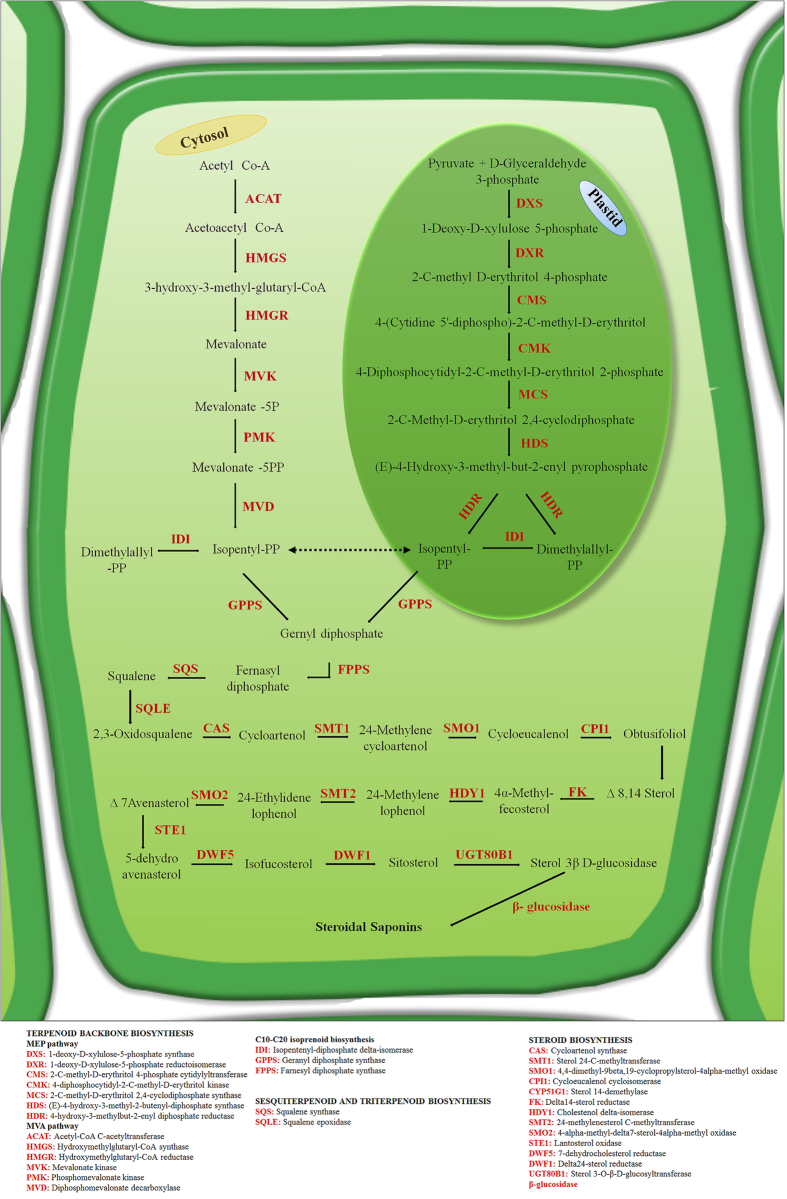
Putative steroidal saponin biosynthesis pathway in *T. govanianum.*

In plants, steroidal saponins are mainly synthesized from lanosterol and cycloartenol *via* cholesterol and sitosterol, respectively^35^,^37^. The genes related to steroidal saponins *via* cholesterol have not been characterized so far, therefore, genes related to steroidal saponins biosynthesis *via* sitosterol were considered in this study, which comprises of three parts; terpenoid backbone, sesquiterpenoid and triterpenoid, and steroid biosynthesis according to KEGG classification (Fig. 6; Supplementary Table S8). Interestingly, all the genes involved in steroidal saponin pathway were identified in the current study. Terpenoid backbone primarily involves the synthesis of DMAPP and IPP from the MVA and MEP pathway and subsequently farnesyl diphosphate (FPP) through sequential head to tail condensation of IPP and DMAPP catalyzed by geranyl diphosphate synthase (GPPS) and farnesyl diphosphate synthase (FPPS)^20^. A total of 16 genes (27 transcripts) involved in terpenoid backbone synthesis were identified in this study. In the sesquiterpenoid and triterpenoid biosynthesis, squalene synthase (SQS) involved in the formation of squalene by condensation of two molecules of FPP, which on oxidation by squalene epoxidase (SQLE) converted into 2,3-oxidosqualene, the branching point between triterpenoid and steroidal saponins^21^. During steroid biosynthesis, the chair-boat-chair-boat conformational change of 2,3-oxidosqualene results in the formation of cycloartenol which ultimately leads to the synthesis of steroidal saponins through various modifications catalyzed by CYPs, isomerase, methyltransferases, reductase, UGTs etc. In total, 14 genes corresponding to 22 transcripts involved in steroid biosynthesis pathway were identified in our study (Fig. 6).

### Expression analysis using qRT-PCR

To study tissue specific expression and validate RNA-Seq data, 29 genes involved in steroidal saponin pathway were selected for the quantitative real-time PCR (qRT-PCR) analysis. In terpenoid backbone biosynthesis, the expression level of six MEP pathway genes, namely 1-deoxy-D-xylulose-5-phosphate synthase (DXS), 1-deoxy-D-xylulose-5-phosphate reductoisomerase (DXR), 2-C-methyl-D-‘kinase (CMK), (E)-4-hydroxy-3-methyl-2-butenyl-diphosphate synthase (HDS) and 4-hydroxy-3-methylbut-2-enyl diphosphate reductase (HDR) was found to be maximum in leaf compared to other tissues, whereas five genes involved in MVA pathway showed variable expression. Acetyl-CoA C-acetyltransferase (ACAT) showed maximum expression in leaf followed by fruit, while hydroxyl methyl glutaryl-CoA reductase (HMGR) was highly expressed in fruit. Phosphomevalonate kinase (PMK) was highly expressed in leaf and fruit, diphosphomevalonate decarboxylase (MVD) was found to be equally expressed in stem, leaf and fruit, while mevalonate kinase (MVK) was highly expressed in rhizome. The FPPS was highly expressed in leaf, while GPPS showed higher expression in rhizome, stem and leaf. In sesquiterpenoid and triterpenoid biosynthesis, SQS and SQLE were highly expressed in leaf and fruit, respectively.

**Figure 7:**
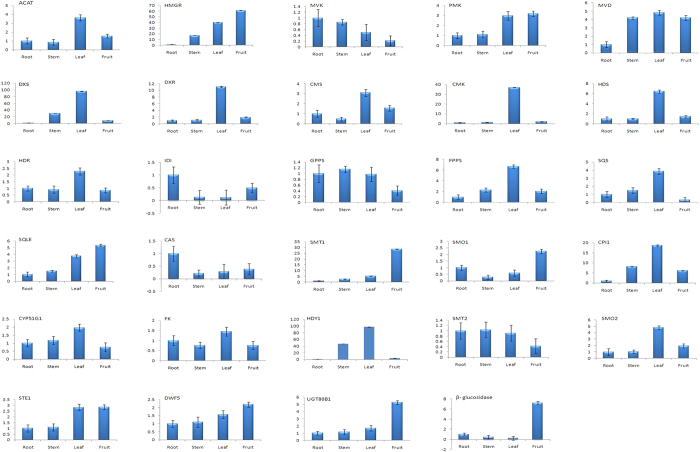
Expression pattern of steroidal saponin biosynthesis pathway genes in different tissues. qRT-PCR analysis was performed using elongation factor 1 alpha (EF1α) as reference gene for normalization. X-axis represents tissues and Y-axis is the relative fold change in gene expression by considering rhizome as control tissue.

All the genes except cycloartenol synthase (CAS), involved in steroid biosynthesis was highly expressed in leaf and fruit tissues. Cycloartenol synthase (CAS) recorded a maximum expression in rhizome. Cycloeucalenol cycloisomerase (CPI1), sterol 14-demethylase (CYP51G1), delta14-sterol reductase (FK), cholestenol delta-isomerase (HYD1) and 4-alpha-methyl-delta7-sterol-4alpha-methyl oxidase (SMO2) were highly expressed in leaf. Sterol 24-C-methyltransferase (SMT1), 4,4-dimethyl-9 beta, 19-cyclopropylsterol-4alpha-methyl oxidase (SMO1), 7-dehydrocholesterol reductase (DWF5), sterol 3-O-β-D-glucosyltransferase (UGT80B1) and β-glucosidase were highly expressed in fruit. Lantosterol oxidase (STE1) recorded comparable expression in leaf and fruit tissues, while, 24-methylenesterol C-methyltransferase (SMT2) was equally expressed in rhizome, stem and leaf (Fig. 7). The overall expression pattern of qRT-PCR was in accordance with the RNA-Seq analysis (Supplementary Figure S2).

## Discussion

A large number of pharmaceutically and industrially important secondary metaboloites are produced by plants through complex biosynthetic pathways. Understanding the biosynthetic pathways and mode of regulation of these compounds in non-model plants, including *T. govanianum* is difficult due to the lack of genomic information. However, the advent of NGS based high throughput transcriptome sequencing has aided to circumvent the difficulties in such plants^38^,^39^. NGS approach has been successfully utilized to elucidate key genes and regulators of complex biosynthetic pathways in a number of non-model plants. *De novo* spatial transcriptome sequencing approach in *T. govanianum*, as performed in this study has also implicated the NGS technology in elucidation of molecular mechanism of complex biosynthesis pathways^40^,^41^,^42^.

Sample wise paired-end reads (37 to 49 millions) obtained in this study was adequate for reliable *de novo* transcriptome characterization and accurate quantification of gene expression pattern^43^. 151,622,376 filtered reads assembled into 69,174 transcripts with an average length of 401 bp obtained in this study are comparable with earlier studies in chickpea (428 bp)^44^ and *Hevea brasiliensis* (436 bp)^45^, while N50 was found to be higher than *Trigonella foenum-graecum* (369 bp)^35^. GC content (46.6%) may be attributed to the ability of *T. govanianum* to adapt in extreme temperatures as GC content play significant role in gene regulation, physical characterization of genome and nucleic acid stability^46^, besides reflecting high quality sequencing run^47^. Interestingly, GC content of *T. govanianum* (46.6%) was higher than Arabidopsis (42.5%)^48^. Despite being a non-model plant, annotation of *T. govanianum* transcripts with multiple public databases successfully assigned putative functions to over 57% of transcripts. Nonetheless, 29,169 (42.82%) transcripts could not be annotated possibly belongs to the untranslated regions or represents the species-specific gene-pool^49^. The assignment of GO terms to a large number of transcripts suggests the presence of diverse gene families in *T. govanianum*. KEGG pathway analysis helps in understanding the biological function and interaction of genes related to the primary and secondary metabolites such that mapping of transcripts with the KEGG database in this study identified all the genes related to steroidal saponin pathway. Based on KOG classification, 31.58% (21845) transcripts were annotated and classified into 25 functional categories, which were comparable to *Curcuma longa* (31.58%)^50^ and higher than *Crocus sativus* (10%)^49^. The role of transcription factors (TFs) as key regulators in controlling gene expression by binding to the promoter of single or multiple genes is well established. Transcription factor families, namely bHLH, bZIP, MYB, MYB-related and WRKY known to facilitate the regulation of various secondary metabolites in plants were well represented in our data. As members of bHLH family, TSAR1 (Triterpene Saponin biosynthesis Activating Regulator1) and TSAR2 regulate triterpene saponin biosynthesis in *Medicago truncatula*^51^, therefore, the identified TFs in this study can be explored as potential regulators of steroidal saponin biosynthesis in *T. govanianum*.

Gene expression analysis has been extensively utilized for the identification of putative regulators of complex molecular pathways by measuring transcriptional levels in different tissues and developmental stages^52^. Identification of large numbers of DEGs in pair-wise comparisons suggests considerable transcriptional differences among tissues of *T. govanianum.* Additionally, important CYPs and UGTs reported to be involved in secondary metabolites biosynthesis including steroidal saponins^23^, showed differential expression among all the tissues supporting spatial metabolite biosynthesis in this plant. The expression analysis revealed that maximum numbers of genes were highly expressed in leaf and fruit tissues, indicating active biosynthesis of steroidal saponin in these tissues. Among the 14 genes of terpenoid backbone synthesis pathway, 11 genes recorded maximum (ACAT, DXS, DXR, CMS, CMK, HDS, HDR and FPPS) and slightly higher/comparable (PMK, MVD and GPPS) expression in leaf indicating that leaf is the primary site for the biosynthesis of steroidal saponins precursors. Higher expression of MEP pathway genes in aerial parts is in accordance with earlier studies^48^. HMGR, a rate limiting enzyme in MVA pathway, involved in the synthesis of phytosterols, carotenoids, gibberellins, triterpenoid and steroidal saponins was found highly expressed in fruit followed by leaf and least expression in rhizome, therefore suggesting its possible role in the early stages of fruit development and defense by producing derivatives of saponins^53^. The higher expression of downstream genes involved in steroidal saponins biosynthesis in leaf (SQS, CPI1, CYP5G1, FK, HDY1 and SMO2) and fruit (SQLE, SMT1, SMO1, DWF5, UGT80B1 and β-glucosidase), also indicated that leaf and fruit tissues are actively involved in the steroidal saponins biosynthesis. The higher expression of ACAT, FPPS, SQS, CPI1, FK and HDY1 in leaf tissue was similar to steroidal sapogenins biosynthesis in *Asparagus racemosus*^54^. As expression pattern of OSCs varies in tissues during plant growth and different developmental stages^55^, we found contrastingly higher expression of CAS in rhizome as compared to other genes.

Global spatial transcriptome analysis of *T. govanianum* suggests that steroidal saponins are synthesized in all the tissues (rhizome, stem, leaf and fruit), with predominance in leaf and fruit. However, the accumulation of steroidal saponins in this species has been reported only in rhizome^16^,^17^, indicating their possible transport to rhizome, similar to ginsenosides biosynthesis in *Panax* spp^56^. As the rate of synthesis and the amount of accumulation of metabolites in different tissue are regulated by many factors such as, rate of transcription, translation, post- transcriptional and post-translational modifications, therefore, correlation cannot be established between biosynthesis and accumulation sites solely with the spatial transcriptome analysis^57^. Additionally, in perennial herbs, the synthesis and accumulation of specialized metabolites in different tissues is greatly influenced by the age and different developmental stages of the plant as reported in *P. ginseng* and *P. quinquefolius*, wherein older plants have higher content of ginsenosides in root, where as leaves accumulates higher metabolite content during early growth stages^56^. To support the tissue specific synthesis, accumulation and transport of steroidal saponins in *T. govanianum*, key findings of this study can be extrapolated with biochemical and histochemical characterization of steroidal saponins in all the tissues during developmental stages under different environmental conditions in an age dependent manner.

## Conclusion

Medicinal plants are vital source of botanical raw drugs for the pharmaceutical industries. We have generated ample dataset through spatial transcriptome sequencing of multiple tissues in the orphan endangered medicinal herb, *T. govanianum.* All the key genes involved in steroidal saponin biosynthesis pathway can be futuristically explored for upscaling of the targeted bioactive molecules at the industrial scale. Additionally, array of CYP450s and UGTs identified in the current study can be good candidates for diversification of bioactive molecules. Maximum expression of key pathway genes and regulatory candidates in leaf and fruit suggests that these can be the site of synthesis of steroidal saponin in *T. govanianum*. Findings from current study will be a pedestal for multi-omics studies in *T. govanianum* and related species for understanding steroidal saponins biosynthesis and its accumulation. Further, comprehensive genomic resource created can be utilized for discovery of the novel genes and functional molecular marker resource for genetic improvement and conservation studies in *T. govanianum*.

## Methods

### Plant materials and RNA isolation

The plant material was collected from its natural habitat at Koksar, Lahaul and Spiti, Himachal Pradesh, India (32°24′03″N, 77°14′24″E) at an altitude of 3631 m. Three genotypes located at a distance of 10 m from each other were randomly considered. Rhizome, stem, leaf and fruit tissues were collected from each genotype. All the samples were frozen immediately in liquid nitrogen and stored at −80 °C till RNA isolation. Total RNA was extracted from individual sample by using iRIS protocol^58^. The concentration of RNA was determined using NanoDrop 2000 spectrophotometer (Thermo Scientific, Lithuania) and integrity was checked on denaturing agarose gel. Equimolar concentration of RNA of three genotypes for each tissue was pooled together for RNA-Seq library preparation to remove the biological biasness.

### Sequencing and *de novo* assembly

RNA-Seq libraries were prepared using Illumina TruSeq RNA sample prep kit v2 (Illumina Inc., USA) according to manufacturer’s instructions. The libraries were quantified using Quant-iT dsDNA Assay Kit, high sensitivity (Invitogen, Eugene, Oregon, USA) and Agilent 2100 Bioanalyzer (Agilent Technologies, USA) was used for library size estimation. Further, for cluster generation, 10 pM of these libraries were loaded onto the flow cell using TruSeq PE Cluster Kit v5 on cluster station (Illumina Inc., USA). Clonally amplified clusters were used for paired-end (PE) (2 × 76) sequencing using Genome Analyzer IIx (Illumina). NGS QC Toolkit^59^ was used to filter raw reads and reads with 75% probability of no error (minimum phred score 20 for each read, and 10 for each base) were utilized for assembly. CLC Genomics Workbench v.6.5 (http://www.clcbio.com) was used for *de novo* transcriptome assembly with default parameters and a minimum transcript length of 200 base pairs.

### Functional annotation and classification

To find the putative functions of assembled transcripts of *T. govanianum*, similarity search using BLASTx^60^ was performed against publicly available protein databases including Arabidopsis proteome (TAIR 10), NCBI non-redundant (nr) and Swiss-Prot with an e-value cut-off of ≤1e^−5^. *T. govanianum* transcripts were classified into three major categories *viz.* biological process, cellular component and molecular function according to Gene Ontology (GO) terms using WEGO software (http://wego.genomics.org.cn/). Transcripts were further functionally categorized into different classes using KOG database (ftp://ftp.ncbi.nih.gov/pub/COG/KOG). The transcription factor (TF) encoding transcripts were identified based on similarity search against Plant Transcription Factor Database (http://planttfdb.cbi.pku.edu.cn). Biochemical pathways were assigned to the transcripts by bi-directional best hit (BBH) method on the KAAS (KEGG Automatic Annotation Server) (http://www.genome.jp/tools/kaas/). CYPs and UGTs present in *T. govanianum* were identified from Swiss-Prot and Arabidopsis Glycosyltransferase Family 1 (http://www.p450.kvl.dk/UGT), respectively.

### Identification of differentially expressed genes

To measure the expression pattern of transcripts in each tissue, high quality reads were mapped onto the final *de novo* assembled transcripts of *T. govanianum* using Tophat2^61^. The expression level for each transcript was measured in terms of Reads Per Kilobase per Million (RPKM) by normalizing read counts according to Mortazavi and co-workers^62^. Further, edgeR package^63^ was used to evaluate the differential gene expression using read counts in the following pair-wise comparisons: leaf vs rhizome, leaf vs fruit, leaf vs stem, fruit vs rhizome, fruit vs stem and stem vs rhizome. Based on statistical analysis, genes having a *p*-value cut off <0.05 and log_2_ fold change ≥2 were considered as differentially expressed genes. The heatmap representing the tissue specific gene expression pattern (log_2_ fold change) for different pathways was generated using Multiple Experiment Viewer (MEV v4.9.0).

### Quantitative Real time PCR (qRT-PCR) analysis

Total RNA was given DNase I (ThermoScientific, *Lithuania*) treatment to remove DNA contamination. First-strand cDNA was synthesized from 2 μg of the total RNA using RevertAid H Minus First Strand cDNA Synthesis Kit (ThermoScientific, *Lithuania*) according to the manufacturer’s instructions followed by 10X dilution. Gene-specific primers (Supplementary Table S8) were designed using BatchPimer3 (http://probes.pw.usda.gov/batchprimer3/). The relative expression of steroidal saponin pathway genes in four tissues was analyzed on StepOnePlus Real-Time PCR Systems (Applied Biosystems, USA) using Power SYBR^®^ Green PCR Master Mix (Applied Biosystems, USA). The qRT-PCR was performed with three technical replicates based on which standard error was calculated. Elongation factor 1 alpha (EF1α) was used as reference gene for establishing equal amount of cDNA in each reaction. The relative gene expression and fold change was calculated using the 2^−ΔΔCT^ method^64^ with rhizome as control tissue.

## Additional Information

**Accession codes:** SRP090722 under the Bioproject- PRJNA345073.

**How to cite this article**: Singh, P. *et al*. Spatial transcriptome analysis provides insights of key gene(s) involved in steroidal saponin biosynthesis in medicinally important herb *Trillium govanianum. Sci. Rep.* **7**, 45295; doi: 10.1038/srep45295 (2017).

**Publisher's note:** Springer Nature remains neutral with regard to jurisdictional claims in published maps and institutional affiliations.

## Supplementary Material

Supplementary Figures and Tables

Supplementary Table S2

Supplementary Table S4

Supplementary Table S5

## References

[b1] Vidyarthi S., Samant S. S. & Sharma P. Dwindling status of Trillium govanianum Wall. ex D. Don-A case study from Kullu district of Himachal Pradesh, India. Journal of Medicinal Plants Research. 7(8), 392–397 (2013).

[b2] Sharma P. & Samant S. S. Diversity, distribution and indigenous uses of medicinal plants in Parbati Valley of Kullu district in Himachal Pradesh, Northwestern Himalaya. Asian J. of Adv. Basic Sci. 2(1), 77–98 (2014).

[b3] Fukuda I. The Origin and Evolution in Trillium. 1. The Origin of the Himalayan Trillium govanianum. Cytologia. 66(1), 105–111 (2001).

[b4] Pellicer J., Kelly L. J., Leitch I. J., Zomlefer W. B. & Fay M. F. A universe of dwarfs and giants: genome size and chromosome evolution in the monocot family Melanthiaceae. New Phytologist. 201(4), 1484–1497 (2014). 24299166 10.1111/nph.12617

[b5] Rani S., Rana J. C. & Rana P. K. Ethnomedicinal plants of Chamba district, Himachal Pradesh, India. Journal of Medicinal Plants Research. 7(42), 3147–3157 (2013).

[b6] Dutt H. C., Bhagat N. & Pandita S. Oral traditional knowledge on medicinal plants in jeopardy among Gaddi shepherds in hills of northwestern Himalaya, J&K, India. Journal of Ethnopharmacology. 168, 337–348 (2015). 25862962 10.1016/j.jep.2015.03.076

[b7] Shah A., Bharati K. A., Ahmad J. & Sharma M. P. New ethnomedicinal claims from Gujjar and Bakerwals tribes of Rajouri and Poonch districts of Jammu and Kashmir, India. Journal of Ethnopharmacology. 166, 119–128 (2015). 25680841 10.1016/j.jep.2015.01.056

[b8] Bhardwaj A. K., Lone P. A., Dar M., Parray J. A. & Shah K. W. Ethnoveterinary medicinal uses of Plants of district Bandipora of Jammu and Kashmir, India. Int. J. Trad. Nat. Med. 2(3), 164–178 (2013).

[b9] Chauhan K. 17 bags of nag chhatri seized at Bindrabani barrier. The Tribune http://www.tribuneindia.com/2012/20120528/himachal.htm (2012).

[b10] Sanjay Kr U. Nagchhatri-A Plant in Peril. Journal of Biodiversity Management & Forestry (2012).

[b11] Samant S. S. & Unit H. Vulnerability Assessment of Biodiversity and Natural Ecosystems in the Selected Sites of Kullu district, Himachal Pradesh.

[b12] Pérez-Labrada K. . ‘Click’ synthesis of triazole-based spirostan saponin analogs. Tetrahedron. 67(40), 7713–7727 (2011).

[b13] Gao X., Sun W., Fu Q. & Niu X. Rapid Identification of Steroidal Saponins in Trillium Tschonoskii Maxim by Ultraperformance Liquid Chromatography Coupled to Electrospray Ionisation Quadrupole Time‐of‐Flight Tandem Mass Spectrometry. Phytochemical Analysis. 26(4), 269–278 (2015). 25808861 10.1002/pca.2560

[b14] Wei J. C. . Steroidal saponins from the rhizomes of Trillium tschonoskii Maxim. Biochemical Systematics and Ecology. 44, 112–116 (2012).

[b15] Hayes P. Y., Lehmann R., Penman K., Kitching W. & De Voss J. J. Steroidal saponins from the roots of *Trillium erectum* (Beth root). Phytochemistry. 70(1), 105–113 (2009). 19091359 10.1016/j.phytochem.2008.10.019

[b16] Ismail M. . Govanoside A, a new steroidal saponin from rhizomes of Trillium govanianum. Steroids. 104, 270–275 (2015). 26505320 10.1016/j.steroids.2015.10.013

[b17] Chauhan N. S. Medicinal and aromatic plants of Himachal Pradesh. Indus Publishing. (1999).

[b18] Chaudhary S. . Elicitation of Diosgenin Production in Trigonellafoenum-graecum (Fenugreek) Seedlings by Methyl Jasmonate. International journal of molecular sciences. 16(12), 29889–29899 (2015). 26694357 10.3390/ijms161226208 PMC4691151

[b19] Sood H., Sharma S., Sharma A., Mehta V., Chauhan R. S. & Malairaman U. Efficient hydroalcoholic extraction for highest diosgenin content from *Trillium govanianum* (nag chhatri) and it’s *in vitro* anticancerous activity. Asian Journal of Pharmaceutical and Clinical Research, 386–392 (2016).

[b20] Naoumkina M. A. . Genomic and coexpression analyses predict multiple genes involved in triterpene saponin biosynthesis in Medicago truncatula. The Plant Cell. 22(3), 850–866 (2010). 20348429 10.1105/tpc.109.073270 PMC2861471

[b21] Abe I. Enzymatic synthesis of cyclic triterpenes. Natural product reports. 24(6), 1311–1331 (2007). 18033581 10.1039/b616857b

[b22] Basyuni M. . Triterpene synthases from the Okinawan mangrove tribe, Rhizophoraceae. FEBS journal. 274(19), 5028–5042 (2007). 17803686 10.1111/j.1742-4658.2007.06025.x

[b23] Seki H., Tamura K. & Muranaka T. P450s and UGTs: key players in the structural diversity of triterpenoid saponins. Plant and Cell Physiology. pcv062 (2015). 10.1093/pcp/pcv062 PMC7107090 25951908

[b24] Wang X., Chen D., Wang Y. & Xie J. De novo transcriptome assembly and the putative biosynthetic pathway of steroidal sapogenins of *Dioscorea composita*. PloS one. 10(4), e0124560 (2015). 25860891 10.1371/journal.pone.0124560 PMC4393236

[b25] Unamba C. I., Nag A. & Sharma R. K. Next Generation Sequencing technologies: The doorway to the unexplored genomics of non-model plants. Frontiers in plant science 6 (2015). 10.3389/fpls.2015.01074 PMC4679907 26734016

[b26] Gene Ontology Consortium. The Gene Ontology (GO) database and informatics resource. Nucleic Acids Research. 32 (suppl 1), D258–D261 (2004). 14681407 10.1093/nar/gkh036 PMC308770

[b27] Kanehisa M. & Goto S. KEGG: kyoto encyclopedia of genes and genomes. Nucleic acids research 28(1), 27–30 (2000). 10592173 10.1093/nar/28.1.27 PMC102409

[b28] VomEndt D., Kijne J. W. & Memelink J. Transcription factors controlling plant secondary metabolism: what regulates the regulators? Phytochemistry. 61(2), 107–114 (2002). 12169302 10.1016/s0031-9422(02)00185-1

[b29] Patra B., Schluttenhofer C., Wu Y., Pattanaik S. & Yuan L. Transcriptional regulation of secondary metabolite biosynthesis in plants. Biochimica et BiophysicaActa (BBA)-Gene Regulatory Mechanisms. 1829(11), 1236–1247 (2013). 10.1016/j.bbagrm.2013.09.006 24113224

[b30] Fujita M., Fujita Y., Noutoshi Y., Takahashi F., Narusaka Y., Yamaguchi-Shinozaki K. & Shinozaki K. Crosstalk between abiotic and biotic stress responses: a current view from the points of convergence in the stress signaling networks. Current opinion in plant biology. 9(4), 436–442 (2006). 16759898 10.1016/j.pbi.2006.05.014

[b31] Mizutani M. & Ohta D. Diversification of P450 genes during land plant evolution. Annual review of plant biology 61, 291–315 (2010). 10.1146/annurev-arplant-042809-112305 20192745

[b32] Lepesheva G. I. & Waterman M. R. Sterol 14α-demethylase cytochrome P450 (CYP51), a P450 in all biological kingdoms. Biochimica et BiophysicaActa (BBA)-General Subjects. 1770(3), 467–477 (2007). 10.1016/j.bbagen.2006.07.018 PMC2324071 16963187

[b33] Jung S. C. . Two ginseng UDP-glycosyltransferases synthesize ginsenoside Rg3 and Rd. Plant and Cell Physiology pcu147 (2014). 10.1093/pcp/pcu147 25320211

[b34] DeBolt S. . Mutations in UDP-glucose: sterol glucosyltransferase in Arabidopsis cause transparent testa phenotype and suberization defect in seeds. Plant physiology. 151(1), 78–87 (2009). 19641030 10.1104/pp.109.140582 PMC2735980

[b35] Vaidya K. . De Novo Transcriptome Sequencing in L. to Identify Genes Involved in the Biosynthesis of Diosgenin. The Plant Genome. 6(2) (2013).

[b36] Sun J. & Zhao Z. Functional features, biological pathways, and protein interaction networks of addiction-related genes. Chemistry & biodiversity 7(5), 1153 (2010). 20491072 10.1002/cbdv.200900319 PMC2939034

[b37] Mehrafarin A. . Bioengineering of important secondary metabolites and metabolic pathways in fenugreek (*Trigonellafoenum-graecum* L.). Journal of Medicinal Plants. 3(35), 1–18 (2010).

[b38] Claros M. G. . Why assembling plant genome sequences is so challenging. Biology. 1(2), 439–459 (2012). 24832233 10.3390/biology1020439 PMC4009782

[b39] Metzker M. L. Sequencing technologies—the next generation. Nature Reviews Genetics. 11(1), 31–46 (2010). 10.1038/nrg2626 19997069

[b40] Gahlan P. . De novo sequencing and characterization of *Picrorhiza kurrooa* transcriptome at two temperatures showed major transcriptome adjustments. BMC Genomics. 13, 126 (2012). 22462805 10.1186/1471-2164-13-126 PMC3378455

[b41] Chakrabarty D. . De novo assembly and characterization of root transcriptome in two distinct morphotypes of vetiver, *Chrysopogon zizaniodes* (L.) Roberty. Scientific Reports. 5 (2015). 10.1038/srep18630 PMC4683516 26679063

[b42] Jayaswall K. . Transcriptome Analysis Reveals Candidate Genes involved in Blister Blight defense in Tea (Camellia sinensis (L) Kuntze). Scientific Reports 6 (2016). 10.1038/srep30412 PMC4964330 27465480

[b43] Vijay N., Poelstra J. W., Künstner A. & Wolf J. B. Challenges and strategies in transcriptome assembly and differential gene expression quantification. A comprehensive in silico assessment of RNA‐seq experiments. Molecular Ecology. 22(3), 620–634 (2013). 22998089 10.1111/mec.12014

[b44] Garg R. . Gene discovery and tissue specific transcriptome analysis in chickpea with passively parallel pyrosequencing and web resource development. Plant Physiology. 156(4) (1661–1678). 10.1104/pp.111.178616 PMC3149962 21653784

[b45] Xia Z. . RNA-Seq analysis and de novo transcriptome assembly of Hevea brasiliensis. Plant Molecular Biology. 77. 299–308 (2011). 21811850 10.1007/s11103-011-9811-z

[b46] Šmarda Petr, . Ecological and evolutionary significance of genomic GC content diversity in monocots. Proceedings of the National Academy of Sciences. 111(39), E4096–E4102 (2014). 10.1073/pnas.1321152111 PMC4191780 25225383

[b47] Ge X., Chen H., Wang H., Shi A. & Liu K. De novo assembly and annotation of Salvia splendens transcriptome using the Illumina platform. PloS one 9(3), e87693 (2014). 24622329 10.1371/journal.pone.0087693 PMC3951189

[b48] Devi K. . Genome wide transcriptome profiling reveals differential gene expression in secondary metabolite pathway of *Cymbopogon winterianus*. Scientific Reports. 6 (2016). 10.1038/srep21026 PMC4753472 26877149

[b49] Jain M., Srivastava P. L., Verma M., Ghangal R. & Garg R. De novo transcriptome assembly and comprehensive expression profiling in Crocus sativus to gain insights into apocarotenoid biosynthesis. Scientific Reports. 6 (2016). 10.1038/srep22456 PMC4776159 26936416

[b50] Annadurai R. S. . De Novo transcriptome assembly (NGS) of Curcuma longa L. rhizome reveals novel transcripts related to anticancer and antimalarial terpenoids. PloS one. 8(2), e56217 (2013). 23468859 10.1371/journal.pone.0056217 PMC3585318

[b51] Goossens A. . The bHLH Transcription Factors TSAR1 and TSAR2 Regulate Triterpene Saponin Biosynthesis in *Medicago truncatula*. Plant physiology. pp-01645 (2015). 10.1104/pp.15.01645 PMC4704604 26589673

[b52] Lovén J. . Revisiting global gene expression analysis. Cell. 151(3), 476–482 (2012). 23101621 10.1016/j.cell.2012.10.012 PMC3505597

[b53] Kim Y. J., Lee O. R., Oh J. Y., Jang M. G. & Yang D. C. Functional analysis of 3-hydroxy-3-methylglutaryl coenzyme a reductase encoding genes in triterpene saponin-producing ginseng. Plant physiology. 165(1), 373–387 (2014). 24569845 10.1104/pp.113.222596 PMC4012596

[b54] Upadhyay S., Phukan U. J., Mishra S. & Shukla R. K. De novo leaf and root transcriptome analysis identified novel genes involved in steroidal sapogenin biosynthesis in *Asparagus racemosus*. BMC Genomics. 15(1), 1 (2014). 25174837 10.1186/1471-2164-15-746 PMC4162912

[b55] Thimmappa R., Geisler K., Louveau T., O’Maille P. & Osbourn A. Triterpene biosynthesis in plants. Annual Review of Plant Biology. 65, 225–257 (2014). 10.1146/annurev-arplant-050312-120229 24498976

[b56] Kim Y. J., Zhang D. & Yang D. C. Biosynthesis and biotechnological production of ginsenosides. Biotechnology advances. 33(6), 717–735 (2015). 25747290 10.1016/j.biotechadv.2015.03.001

[b57] Feussner I. & Polle A. What the transcriptome does not tell—proteomics and metabolomics are closer to the plants’ patho-phenotype. Current opinion in plant biology 26, 26–31 (2015). 26051215 10.1016/j.pbi.2015.05.023

[b58] Ghawana S. . An RNA isolation system for plant tissues rich in secondary metabolites. BMC Research Notes. 4(1), 85 (2011). 21443767 10.1186/1756-0500-4-85 PMC3079660

[b59] Patel R. K. & Jain M. NGS QC Toolkit: a toolkit for quality control of next generation sequencing data. PloS one 7(2), e30619 (2012). 22312429 10.1371/journal.pone.0030619 PMC3270013

[b60] Altschul S. F., Gish W., Miller W., Myers E. W. & Lipman D. J. Basic local alignment search tool. Journal of Molecular Biology. 215(3), 403–410 (1990). 2231712 10.1016/S0022-2836(05)80360-2

[b61] Kim D. . TopHat2: accurate alignment of transcriptomes in the presence of insertions, deletions and gene fusions. Genome Biology. 14(4), 1 (2013). 10.1186/gb-2013-14-4-r36 PMC4053844 23618408

[b62] Mortazavi A., Williams B. A., McCue K., Schaeffer L. & Wold B. Mapping and quantifying mammalian transcriptomes by RNA-Seq. Nature Methods. 5(7), 621–628 (2008). 18516045 10.1038/nmeth.1226 PMC13303166

[b63] Robinson M. D., McCarthy D. J. & Smyth G. K. edgeR: a Bioconductor package for differential expression analysis of digital gene expression data. Bioinformatics. 26(1), 139–140 (2010). 19910308 10.1093/bioinformatics/btp616 PMC2796818

[b64] Livak K. J. & Schmittgen T. D. Analysis of relative gene expression data using real-time quantitative PCR and the 2−ΔΔCT method. Methods. 25(4), 402–408 (2001). 11846609 10.1006/meth.2001.1262

