# Supplementary material for: Spatial transcriptome analysis provides insights of key gene(s) involved in steroidal saponin biosynthesis in medicinally important herb Trillium govanianum
Source: Sci Rep. 2017 Mar 28;7:45295. doi: 10.1038/srep45295 (PMC5368571; doi:10.1038/srep45295)
Supplement: Supplementary Figures and Tables [file srep45295-s1.pdf]

Supplementary Information: SREP-16-41203

Spatial transcriptome analysis provides insights of key gene(s) involved in steroidal saponin biosynthesis in medicinally important herb *Trillium govanianum*

Pradeep Singh, Gagandeep Singh, Abhishek Bhandawat, Gopal Singh, Rajni Parmar, Romit Seth, Ram Kumar Sharma*

Biotechnology Department, CSIR-Institute of Himalayan Bioresource Technology, Palampur, Himachal Pradesh, India, 176061

***Correspondence:**

Dr Ram Kumar Sharma
Biotechnology Division
CSIR-Institute of Himalayan Bioresource Technology
Palampur (H.P)
India
Email: rksharma.ihbt@gmail.com, ramsharma@ihbt.res.in

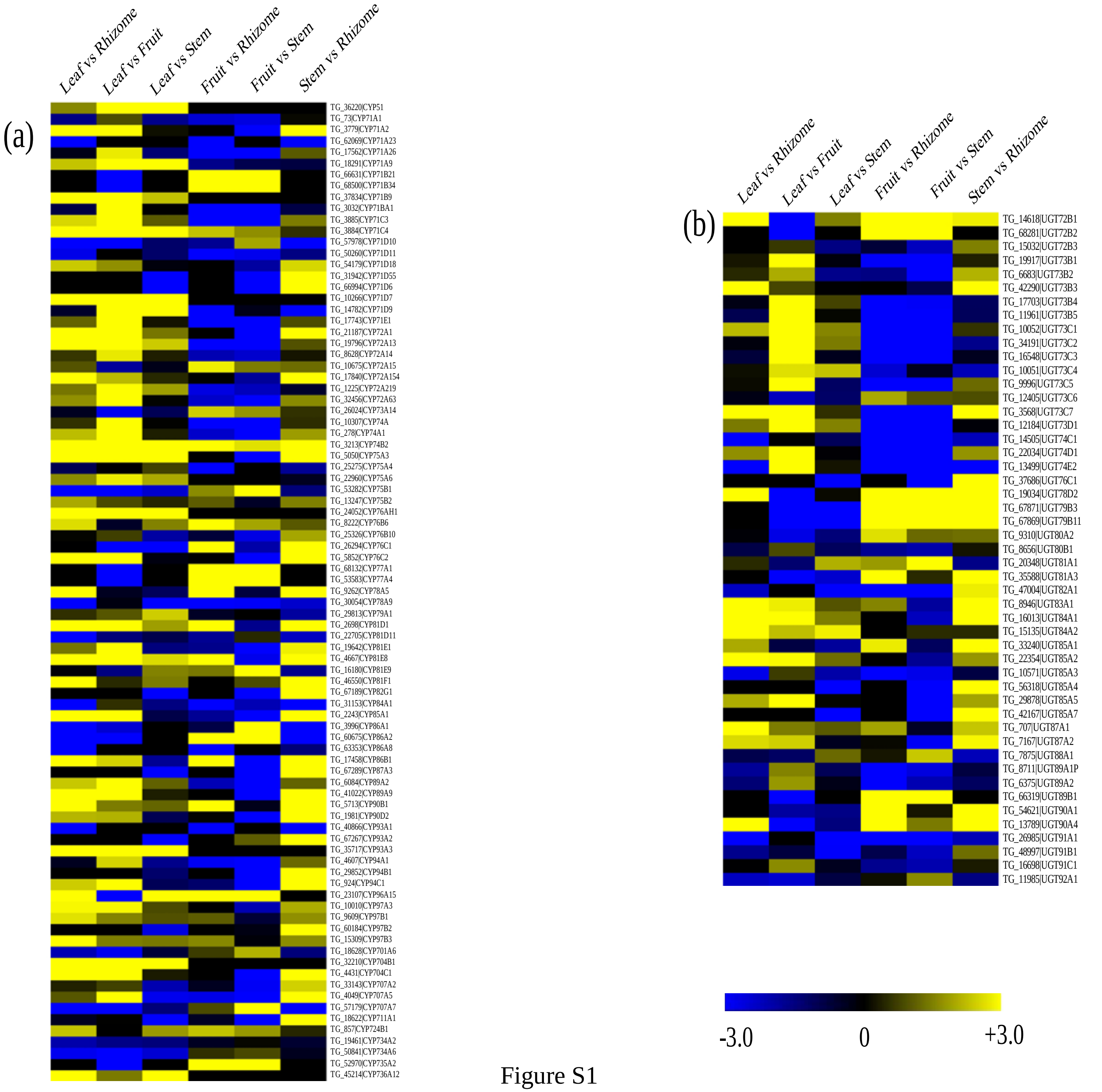
(a)
Leaf vs Rhizome
Leaf vs Fruit
Leaf vs Stem
Fruit vs Rhizome
Fruit vs Stem
Stem vs Rhizome
TG_36220|CYP51
TG_73|CYP71A1
TG_3779|CYP71A2
TG_62069|CYP71A23
TG_17562|CYP71A26
TG_18291|CYP71A9
TG_66631|CYP71B21
TG_68500|CYP71B34
TG_37834|CYP71B9
TG_3032|CYP71BA1
TG_3885|CYP71C3
TG_3884|CYP71C4
TG_57978|CYP71D10
TG_50260|CYP71D11
TG_54179|CYP71D18
TG_31942|CYP71D55
TG_66994|CYP71D6
TG_10266|CYP71D7
TG_14782|CYP71D9
TG_17743|CYP71E1
TG_21187|CYP72A1
TG_19796|CYP72A13
TG_8628|CYP72A14
TG_10675|CYP72A15
TG_17840|CYP72A154
TG_1225|CYP72A219
TG_32456|CYP72A63
TG_26024|CYP73A14
TG_10307|CYP74A
TG_278|CYP74A1
TG_3213|CYP74B2
TG_5050|CYP75A3
TG_25275|CYP75A4
TG_22960|CYP75A6
TG_53282|CYP75B1
TG_13247|CYP75B2
TG_24052|CYP76AH1
TG_8222|CYP76B6
TG_25326|CYP76B10
TG_26294|CYP76C1
TG_5852|CYP76C2
TG_68132|CYP77A1
TG_53583|CYP77A4
TG_9262|CYP78A5
TG_30054|CYP78A9
TG_29813|CYP79A1
TG_2698|CYP81D1
TG_22705|CYP81D11
TG_19642|CYP81E1
TG_4667|CYP81E8
TG_16180|CYP81E9
TG_46550|CYP81F1
TG_67189|CYP82G1
TG_31153|CYP84A1
TG_2243|CYP85A1
TG_3996|CYP86A1
TG_60675|CYP86A2
TG_63353|CYP86A8
TG_17458|CYP86B1
TG_67289|CYP87A3
TG_6084|CYP89A2
TG_41022|CYP89A9
TG_5713|CYP90B1
TG_1981|CYP90D2
TG_40866|CYP93A1
TG_67267|CYP93A2
TG_35717|CYP93A3
TG_4607|CYP94A1
TG_29852|CYP94B1
TG_924|CYP94C1
TG_23107|CYP96A15
TG_10010|CYP97A3
TG_9609|CYP97B1
TG_60184|CYP97B2
TG_15309|CYP97B3
TG_18628|CYP701A6
TG_32210|CYP704B1
TG_4431|CYP704C1
TG_33143|CYP707A2
TG_4049|CYP707A5
TG_57179|CYP707A7
TG_18622|CYP711A1
TG_857|CYP724B1
TG_19461|CYP734A2
TG_50841|CYP734A6
TG_52970|CYP735A2
TG_45214|CYP736A12
(b)
Leaf vs Rhizome
Leaf vs Fruit
Leaf vs Stem
Fruit vs Rhizome
Fruit vs Stem
Stem vs Rhizome
TG_14618|UGT72B1
TG_68281|UGT72B2
TG_15032|UGT72B3
TG_19917|UGT73B1
TG_6683|UGT73B2
TG_42290|UGT73B3
TG_17703|UGT73B4
TG_11961|UGT73B5
TG_10052|UGT73C1
TG_34191|UGT73C2
TG_16548|UGT73C3
TG_10051|UGT73C4
TG_9996|UGT73C5
TG_12405|UGT73C6
TG_3568|UGT73C7
TG_12184|UGT73D1
TG_14505|UGT74C1
TG_22034|UGT74D1
TG_13499|UGT74E2
TG_37686|UGT76C1
TG_19034|UGT78D2
TG_67871|UGT79B3
TG_67869|UGT79B11
TG_9310|UGT80A2
TG_8656|UGT80B1
TG_20348|UGT81A1
TG_35588|UGT81A3
TG_47004|UGT82A1
TG_8946|UGT83A1
TG_16013|UGT84A1
TG_15135|UGT84A2
TG_33240|UGT85A1
TG_22354|UGT85A2
TG_10571|UGT85A3
TG_56318|UGT85A4
TG_29878|UGT85A5
TG_42167|UGT85A7
TG_707|UGT87A1
TG_7167|UGT87A2
TG_7875|UGT88A1
TG_8711|UGT89A1P
TG_6375|UGT89A2
TG_66319|UGT89B1
TG_54621|UGT90A1
TG_13789|UGT90A4
TG_26985|UGT91A1
TG_48997|UGT91B1
TG_16698|UGT91C1
TG_11985|UGT92A1
-3.0
0
+3.0

Figure S1

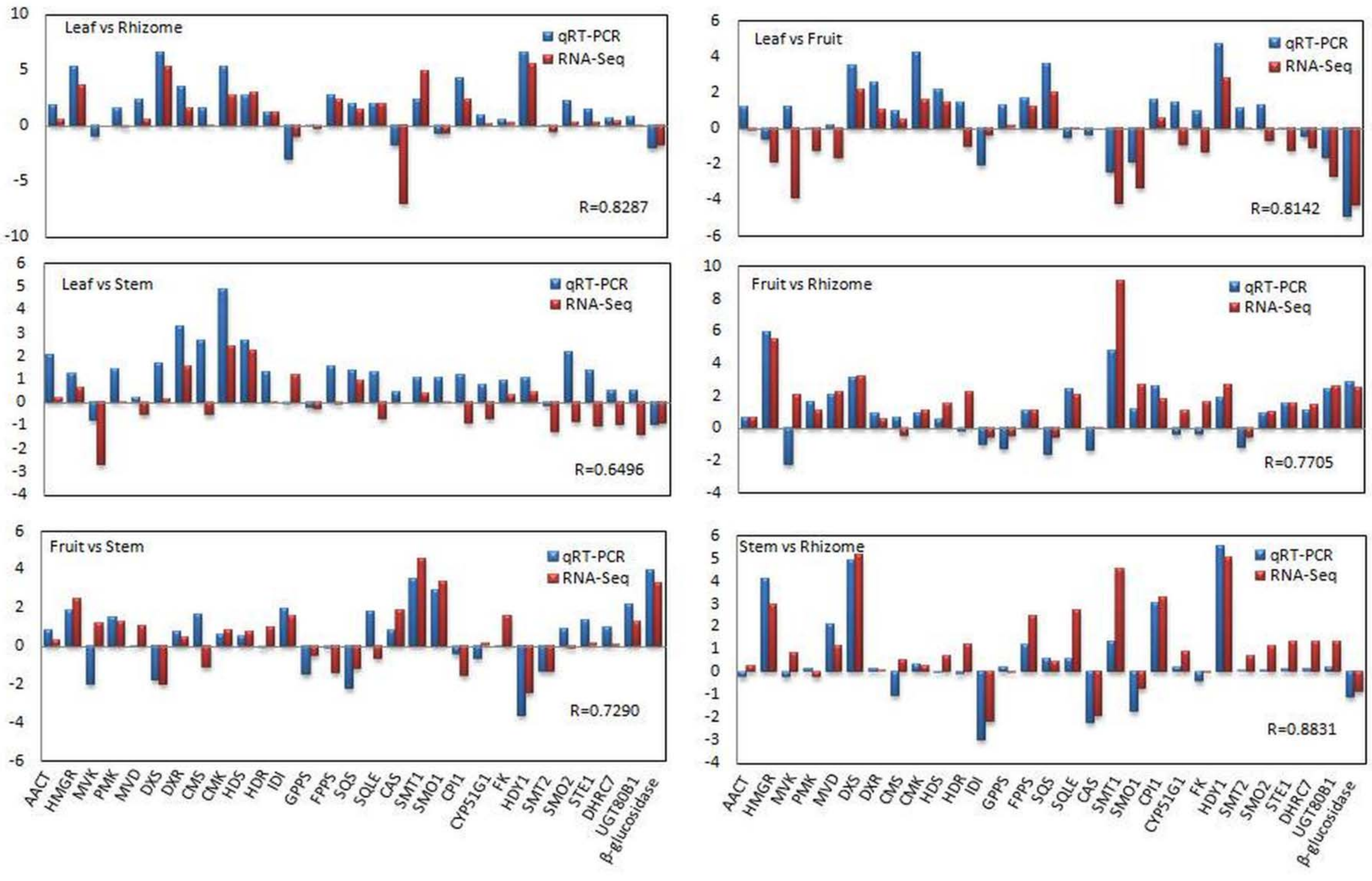
Leaf vs Rhizome
qRT-PCR
RNA-Seq
R=0.8287
Leaf vs Fruit
R=0.8142
Leaf vs Stem
R=0.6496
Fruit vs Rhizome
R=0.7705
Fruit vs Stem
R=0.7290
Stem vs Rhizome
R=0.8831
AACT
HMGR
MVK
PMK
MVD
DXS
DXR
CMS
CMK
HDS
HDR
IDI
GPPS
FPPS
SQS
SQLE
CAS
SMT1
SMO1
CPI1
CYP51G1
FK
HDY1
SMT2
SMO2
STE1
DHRC7
UGT80B1
β-glucosidase

Figure S2

Supplementary Table S1: **Summary of *de novo* assembly generated from *T. govanianum*.**

Categories	Characteristics
Number of paired-end raw reads	173,974,146
N50 length	412
Minimum length (bp)	174
Maximum length (bp)	35,947
Average length (bp)	401
Total Contigs	69,174

Supplementary Table S3: Classification and number of transcripts related to transcription factors families.

S. No.	Family	Genes	Transcripts
1.	bHLH	250	1036
2.	MYB_related	188	662
3.	NAC	155	628
4.	FAR1	125	559
5.	bZIP	147	516
6.	B3	82	499
7.	WRKY	128	484
8.	C2H2	166	415
9.	ERF	140	410
10.	C3H	180	403
11.	MYB	128	386
12.	G2-like	83	276
13.	GRAS	89	243
14.	HD-ZIP	79	213
15.	M-type	44	181
16.	Trihelix	65	176
17.	GATA	52	173
18.	TCP	34	158
19.	HB-other	45	156
20.	HSF	44	143
21.	NF-YB	32	136
22.	ARF	69	135
23.	MIKC	34	134
24.	TALE	41	107
25.	LBD	37	106
26.	E2F/DP	25	103
27.	YABBY	13	97
28.	S1Fa-like	16	89
29.	GeBP	17	88
30.	SBP	35	87
31.	Nin-like	22	78
32.	Dof	34	72
33.	STAT	5	65
34.	NF-YA	18	63
35.	BES1	23	62
36.	NF-YC	26	62
37.	WOX	13	60
38.	AP2	22	48
39.	CAMTA	24	46
40.	NF-X1	9	45
41.	CO-like	23	40
42.	CPP	16	40
43.	BBR-BPC	14	37
44.	SRS	8	37
45.	EIL	14	34
46.	ARR-B	25	33
47.	DBB	15	33
48.	ZF-HD	16	27
49.	LSD	11	25
50.	VOZ	7	23
51.	HRT-like	6	19
52.	GRF	14	18
53.	RAV	7	16
54.	Whirly	7	15
55.	HB-PHD	3	7
56.	LFY	1	1
57.	NZZ/SPL	1	1
58.	SAP	1	1

Supplementary Table S6. Pathways and number of transcripts related to secondary metabolite biosynthesis in *T. govanianum*

S. No.	Pathway ID	Secondary metabolites biosynthesis pathways	KO	Transcripts
1.	Ko00254	Aflatoxin biosynthesis	1	1
2.	Ko00942	Anthocyanin biosynthesis	1	1
3.	Ko00965	Betalain biosynthesis	1	1
4.	Ko00905	Brassinosteroid biosynthesis	4	4
5.	Ko00524	Butirosin and neomycin biosynthesis	2	3
6.	Ko00332	Carbapenem biosynthesis	2	2
7.	Ko00906	Carotenoid biosynthesis	14	18
8.	Ko00904	Diterpenoid biosynthesis	7	8
9.	Ko00944	Flavone and flavonol biosynthesis	3	4
10.	Ko00941	Flavonoid biosynthesis	12	13
11.	Ko00966	Glucosinolate biosynthesis	2	2
12.	Ko00901	Indole alkaloid biosynthesis	2	2
13.	Ko00950	Isoquinoline alkaloid biosynthesis	8	9
14.	Ko00261	Monobactam biosynthesis	6	9
15.	Ko00902	Monoterpenoid biosynthesis	3	3
16.	Ko00401	Novobiocin biosynthesis	3	4
17.	Ko00311	Penicillin and cephalosporin biosynthesis	1	1
18.	Ko00940	Phenylpropanoid biosynthesis	18	32
19.	Ko00523	Polyketide sugar unit biosynthesis	1	1
20.	Ko00909	Sesquiterpenoid and triterpenoid biosynthesis	2	2
21.	Ko00100	Steroid biosynthesis	18	23
22.	Ko00945	Stilbenoid, diarylheptanoid and gingerol biosynthesis	5	8
23.	Ko00521	Streptomycin biosynthesis	6	12
24.	Ko00900	Terpenoid backbone biosynthesis	33	47
25.	Ko00253	Tetracycline biosynthesis	3	3
26.	Ko00960	Tropane, piperidine and pyridine alkaloid biosynthesis	9	12
27.	Ko00908	Zeatin biosynthesis	4	7

Supplementary Table S7: List of expression primer sets of selected transcripts used for Real time-PCR analysis.

S. No.	Gene Symbol	Transcript ID	Forward 5'-3'	Reverse 5'-3'
1.	ACAT	TG_1384	GTGCCCAAATCGAGATCCT	CTTGCGGCACAGAGTATTCA
2.	HMGR	TG_7012	GGACGTGATTAGCATCTCAGG	TCACCACCTGCTCTTTGATG
3.	MVK	TG_27721	TCAGCTGGTGAATAAATGTGCT	GTCTCACCTGACCGGAACAT
4.	PMK	TG_12165	CTTTGCCTCCATTGATGACC	TGTCTCCATGGATCTCTGAGG
5.	MVD	TG_4239	CATTAGGCCCTGCATCAAAT	CAAGCCCGCCAATATTCTAC
6.	DXS	TG_13335	TTGGCAAAGGGAGGATACTG	CATCCCATGCTGCTCTACAA
7.	DXR	TG_1064	AGCCGCAATAGAGTCTGGAA	TCGGAATCAGCTGGCAGTAT
8.	CMS	TG_22136	CCCAAAGTGTTCTCCTGTCG	AGAGATGGTTGGCTGAATGG
9.	CMK	TG_16822	TGCCTTGTGAGAAAGGAAGC	TCAGGAAGCGGAAGCACTAT
10.	HDS	TG_241	CCATTCAAGGATCTGGCAAC	GTCAGAACGCCCATGCTTAT
11.	HDR	TG_29033	AGCGGAGTCTTACGGGTTCT	GACGGTTGGGTTGTGGATAA
12.	IDI	TG_28659	GCTTTATGCGCCAGTTGAAT	TCTCACCGCAAACGGAAC
13.	GPPS	TG_15477	GTCAACATCGCTTGGGAAAG	AAGCCACGGTCAACAACTTC
14.	FPPS	TG_20699	GGGCAGATGTTGGATTTGAT	GCAACCGGAAGGTAGAAGG
15.	SQS	TG_2891	TTGATGCATGCTGAAGATTG	TGTAGCATTGCGCTAGTGTTC
16.	SQLE	TG_10901	GCCGGTTTCCTCTACGATAA	ACACCTCCGAGGCTCAGATA
17.	CAS	TG_61680	AGCCAATTGTGGGACACAG	GCCAGGACAGTTGTCAAGAA
18.	SMT1	TG_12295	CCAGTGGCAAGTACCATGAA	ACGGGCTTCCAGACATAAGA
19.	SMO1	TG_30966	CCCTTCTATGGAGGTGCAGA	GCCCTTGTCAGTTCCGTAAA
20.	CPI1	TG_15731	ATGAACAATGTGCCTCATGC	TGATTGTGGTAAGTCAGCAACA
21.	CYP51G1	TG_4589	AGGTGATGGAGCTCGTGTG	GACGACATGCTCCAATGCT
22.	FK	TG_15784	GCACCTCTCCTCGTCTCTTCT	CTATTGCTCGCGCTTTCATT
23.	HDY1	TG_1045	GCCGGTATGATAATCCACGA	TGGATGCGCTGTTTACTTTG
24.	SMT2	TG_2945	ACGATCACGGCAGACAAAGT	TGTAGAAGGTGTCGACGAAGG
25.	SMO2	TG_5104	GAACACTGTGGACGTGCTTG	TTGCCACACTGGACTGTCAT
26.	STE1	TG_5587	AGTTGCGATGAAGGCTATGC	TACATGGGCCAACCAACTTC
27.	DWF5	TG_27363	ATCCCGTAAATCTCGGTTCC	GGCATTTCCCATTTGTTCTG
28.	UGT80B1	TG_9310	CGGCTCTAAGACCAGCAGAA	TTGGCAGAACCCAGAGATTC
29.	β-glucosidase	TG_4604	CAGCCAGAGGGTTCTCAAAG	GACGATCTGACTGCCCTTGT

The PCR amplification program was as follows: 95°C for 10min; 40 cycles of 95°C for 30s, 58°C for 30s and 72°C for 30s; followed by a melting-curve program of 95°C for 1min, 60°C for 30s and 95°C for 30s.

Supplementary Table S8: The number of transcripts related to Steroidal Saponin biosynthesis.

S. No.	Gene	Gene Symbol	KO	Transcripts
Terpenoid backbone biosynthesis	Acetyl-CoA C-acetyltransferase	ACAT	K00626	1
	Hydroxymethylglutaryl-CoA synthase	HMGS	K01641	2
	Hydroxymethylglutaryl-CoA reductase	HMGR	K00021	1
	Mevalonate kinase	MVK	K00869	1
	Phosphomevalonate kinase	PMK	K00938	1
	Diphosphomevalonate decarboxylase	MVD	K01597	1
	1-deoxy-D-xylulose-5-phosphate synthase	DXS	K01662	1
	1-deoxy-D-xylulose-5-phosphate reductoisomerase	DXR	K00099	1
	2-C-methyl-D-erythritol 4-phosphate cytidylyltransferase	CMS	K00991	1
	4-diphosphocytidyl-2-C-methyl-D-erythritol kinase	CMK	K00919	1
	2-C-methyl-D-erythritol 2,4-cyclodiphosphate synthase	MCS	K01770	3
	(E)-4-hydroxy-3-methyl-2-butenyl-diphosphate synthase	HDS	K03526	5
	4-hydroxy-3-methylbut-2-enyl diphosphate reductase	HDR	K03527	3
	Isopentenyl-diphosphate delta-isomerase	IDI	K01823	3
	Geranyl diphosphate synthase	GPPS	K14066	1
	Farnesyl diphosphate synthase	FPPS	K00787	1
Sesquiterpenoid and triterpenoid biosynthssis	Squalene synthase	SQS	K00801	1
	Squalene epoxidase	SQLE	K00511	1
Steroidal saponinbuosynthesis	Cycloartenol synthase	CAS	K01853	1
	Sterol 24-C-methyltransferase	SMT1	K00559	1
	4,4-dimethyl-9beta,19-cyclopropylsterol-4alpha-methyl oxidase	SMO1	K14423	1
	Cycloeucalenolcycloisomerase	CPI1	K08246	1
	Sterol 14-demethylase	CYP51G1	K05917	1
	Delta14-sterol reductase	FK	K00222	1
	Cholestenol Delta-isomerase	HDY1	K01824	2
	24-methylenesterol C-methyltransferase	SMT2	K08242	1
	4-alpha-methyl-delta7-sterol-4alpha-methyl oxidase	SMO2	K14424	1
	Lantosterol Oxidase	STE1	K00227	1
	7-dehydrocholesterol reductase	DWF5	K00213	1
	Delta24-sterol reductase	DWF1	K09828	3
	Sterol 3-O-β-D-glucosyltransferase	UGT80B1	K05841	1
	β-glucosidase	β-glucosidase	K05349	3
		β-glucosidase	K01188	1

Supplementary Figure legends

Supplementary Figure S1: Heatmap showing tissue-specific gene expression patterns in *T. govanianum*;(a) UGTs and (b) CYP450.

Supplementary Figure S2: Correlation analysis between qRT-PCR and RNA-seq data of steroidal saponin biosynthesis pathway genes using log2 FC.

Supplementary Table Titles

Supplementary Table S1: Summary of *de novo* assembly generated from *T. govanianum.*

Supplementary Table S2: Functional annotation of T. govanianum transcripts with various public databases. **(Submitted as Supplementary dataset)**

Supplementary Table S3: Classification and number of transcripts related to transcription factors families.

Supplementary Table S4: CYPs Classification: Details of CYPs family, sub-family, genes and contig IDs. **(Submitted as Supplementary dataset)**

Supplementary Table S5: Global and tissue specific expression of cytochrome P450s (CYPs, s.no. 1), uridine diphosphate-glycosyltransferases (UGTs, s.no. 2), secondary metabolite biosynthesis pathways (s.no. 3) and steroidal saponin biosynthesis pathway (s.no. 4) in *T. govanianum*. Tissue wise expression of different categories was depicted with green colour (Leaf), Stem (Blue), Rhizome (yellow), Fruit (red). **(Submitted as Supplementary dataset)**

Supplementary Table S6. Pathways and number of transcripts related to secondary metabolite biosynthesis in *T. govanianum.*

Supplementary Table S7: List of expression primer sets of selected transcripts used for Real time-PCR analysis.
Supplementary Table S8: The number of transcripts related to Steroidal Saponin biosynthesis.
